# IgG Subclass Switch in Volunteers Repeatedly Immunized with the Full-Length *Plasmodium falciparum* Merozoite Surface Protein 1 (MSP1)

**DOI:** 10.3390/vaccines12020208

**Published:** 2024-02-17

**Authors:** Veronika Rathay, Kristin Fürle, Viktoria Kiehl, Anne Ulmer, Michael Lanzer, Richard Thomson-Luque

**Affiliations:** 1 Parasitology, Centre for Infectious Diseases, University Hospital Heidelberg, Medical Faculty, Heidelberg University, 69120 Heidelberg, Germany; 2 Sumaya-Biotech GmbH & Co. KG, 69115 Heidelberg, Germany

**Keywords:** malaria, vaccines, *Plasmodium falciparum*, MSP1, IgG subclass

## Abstract

Vaccines are highly effective tools against infectious diseases and are also considered necessary in the fight against malaria. Vaccine-induced immunity is frequently mediated by antibodies. We have recently conducted a first-in-human clinical trial featuring SumayaVac-1, a malaria vaccine based on the recombinant, full-length merozoite surface protein 1 (MSP1_FL_) formulated with GLA-SE as an adjuvant. Vaccination with MSP1_FL_ was safe and elicited sustainable IgG antibody titers that exceeded those observed in semi-immune populations from Africa. Moreover, IgG antibodies stimulated various Fc-mediated effector mechanisms associated with protection against malaria. However, these functionalities gradually waned. Here, we show that the initial two doses of SumayaVac-1 primarily induced the cytophilic subclasses IgG1 and IgG3. Unexpectedly, a shift in the IgG subclass composition occurred following the third and fourth vaccinations. Specifically, there was a progressive transition to IgG4 antibodies, which displayed a reduced capacity to engage in Fc-mediated effector functions and also exhibited increased avidity. In summary, our analysis of antibody responses to MSP1_FL_ vaccination unveils a temporal shift towards noninflammatory IgG4 antibodies. These findings underscore the importance of considering the impact of IgG subclass composition on vaccine-induced immunity, particularly concerning Fc-mediated effector functions. This knowledge is pivotal in guiding the design of optimal vaccination strategies against malaria, informing decision making for future endeavors in this critical field.

## 1. Introduction

The current resurgence of malaria [1,2] accentuates the need for an effective malaria vaccine [3]. Such vaccines would ideally target multiple developmental forms in the complex life cycle of malaria parasites, including the incoming sporozoites, the liver stages, the disease-causing asexual blood stages and the transmissible gametocyte stages. Thus far, great progress has only been made in the development of pre-erythrocytic vaccines against *Plasmodium falciparum*, the protozoan parasite that causes the most virulent form of malaria in humans. This includes RTS,S (Mosquirix) [4,5], the first approved malaria vaccine, and R21 [6,7], which was recently recommended by the World Health Organization (WHO). While the development of pre-erythrocytic vaccines will hopefully prevent thousands of deaths every year, especially of African children under the age of five, there is a caveat though. Pre-erythrocytic vaccines do not prevent blood stage development and every parasite that escapes the vaccine cover will infect erythrocytes and eventually cause life-threatening disease [8]. It is therefore advisable to add a blood stage component to pre-erythrocytic vaccines.

The generation of robust levels of antibodies plays a crucial role towards life-lasting immunity against infectious diseases through natural exposure to pathogens [9]. As for malaria, passive immunization studies have demonstrated that IgG antibodies play a key role in controlling a blood-stage infection [10]. Furthermore, people living in malaria-endemic regions can acquire a semi-immune status through life-long exposure to the parasite, which is associated with the progressive generation of memory B cells and long-lasting plasma cells [11], thereby rendering re-infections asymptomatic and non-life threatening [12]. However, life-lasting and sterilizing protection against malaria is rarely achieved [10].

Long-lasting immunity can be induced by vaccination as demonstrated in other systems, such as tetanus and diphtheria [13]. Attempts to elicit a robust long-lasting humoral immunity to malaria, by safeguarding persisting antibody responses, have not yet been successful. Antibody titers tend to wane rapidly following immunization, as exemplified by vaccination with RTS,S [14]. Recently, the same phenomenon has been massively experienced with mRNA vaccines against SARS-CoV-2 [15], with loss of protection occurring several months after vaccination, probably through the generation of immune tolerance [16].

Sero-epidemiological studies and humoral responses to vaccines have typically focused on measuring total IgG antibodies, but the examination of specific IgG subclasses (IgG1, IgG2, IgG3, and IgG4) is less common. Understanding the quantitative differences between IgG subclasses and their distinct biological and functional properties in natural infections and, importantly, through the course of vaccination, may aid in unveiling the cause underlying the reduction in vaccine efficacy over time and may further aid vaccine design. For example, the importance of cytophilic antibodies in the natural control of HIV infection has been linked to the production and persistence of elevated levels of IgG1 and IgG3 subclasses [16]. As for vaccines, a comprehensive analysis of the longitudinal evolution of all four IgG subclasses in response to SARS-CoV-2 mRNA vaccination [15] has recently shown that the predominant IgG subclasses induced were IgG1 and IgG3. However, there was an increase in anti-spike IgG4 antibodies months after receiving the second mRNA immunization. This late increase in IgG4 is paralleled by the loss in Fc-mediated effector functions, which are generally attributed to cytophilic IgG1 and IgG3 antibodies [17].

IgG1 and IgG3 cytophilic subclasses are also recognized as protective antibodies against *Plasmodium* spp. infections [18,19,20,21,22,23,24,25,26]. IgG1 and IgG3 exhibit a strong affinity for a wide range of Fc receptors on various immune cells [27]. They mediate protection against malaria through opsonization, leading to complement-mediated lysis [28], phagocytosis [26,29,30,31], respiratory burst and antibody-dependent cellular inhibition [32,33]. In comparison, IgG2 and IgG4’s primary function lies in neutralization [27] but are considered non-protective antibodies against malaria [19,23,27,31,34] due to the absence of affinity for complement and the limited engagement with Fc receptors [27]. In the case of RTS,S, analyses on the significance of antibody subclasses targeting the circumsporozoite protein (CSP) revealed that the induced IgG subclass profile played a critical role in immune protection, with anti-CSP-specific IgG1 and IgG3 being associated with protection [35].

**Figure 1:**
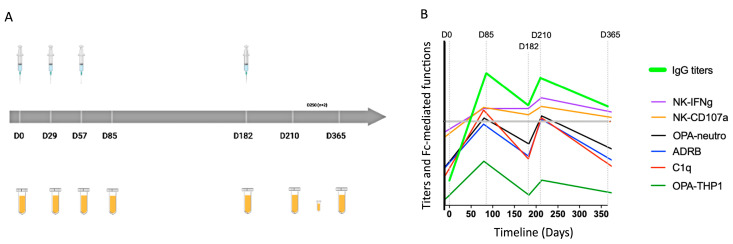
First-in-human phase Ia clinical trial of MSP1_FL_. (**A**) Representative scheme of the immunization study design and sample collection timepoints. Blood samples were collected on the specified days for subsequent immunological analysis. A safety follow-up timepoint was planned on day 365 (except for 2 individuals who could only be followed up to day 270). (**B**) Compilation of anti-MSP1_FL_-specific IgG titers and IgG Fc-mediated effector functions at days 0, 85, 182, 210 and 365 through the vaccination period. IgG titers were measured in an ELISA assay [36]. Fc-mediated effector functions were measured through an array of functional in vitro assays [37]. These assays involved C1q complement fixation (Abc’), opsonic phagocytosis by THP1 cells (OPA-THP1), and neutrophils (neutrophil OPA), respiratory burst of neutrophils (ADRB) and NK cell stimulation leading to degranulation (CD107a) and IFNγ production (IFNγ). A significant loss in functionality was observed between day 210 and the follow-up timepoint at day 365, except for NK-assays CD107a and IFNγ. Grey line represents the reference level for IgG titers and all functionalities from pooled sera from Kisumu, Kenya (WHO reference standard).

We have previously conducted a first-in-human, dose escalation, phase Ia clinical trial of the full-length merozoite surface protein 1 (MSP1_FL_) combined with the GLA-SE adjuvant (henceforth termed Sumaya-Vac1) as a vaccine against the disease-causing blood stages of *P. falciparum* in naïve individuals [36]. All volunteers received 3 monthly immunizations on days 0, 29, and 57, with an optional booster being administered to some individuals after cohort unblinding on day 182 (Figure 1A). All vaccinees seroconverted with anti-MSP1_FL_-specific IgG titers remaining high after one year. IgG titers exceeded those of a pool of sera from malaria-pre-exposed adult individuals from Kisumu, Kenya (WHO reference level), as well as titers of pooled sera from adult individuals from Nouna, Burkina Faso, until the end of the study [36]. Several in vitro measured Fc receptor-mediated functions elicited by IgG from vaccinees exceeded those observed for Kenyan individuals right after immunizations, but, in general, waned gradually under the levels observed for the semi-immune population [37]. The study vaccination and sample collection timepoints, as well as the IgG titers and functionality previously reported, are schematically presented in Figure 1.

Here, we report a longitudinal analysis of the IgG antibody subclass responses in the context of this phase Ia trial of SumayaVac-1 to unveil the distinct long-term dynamics between IgG subclass titers and functionality after vaccination with MSP1_FL_. We describe a shift in the composition of anti-MSP1_FL_-specific IgG subclasses. At the end of the study, reduced levels of the multifunctional IgG3 led to increased levels of IgG4 characterized by higher antibody avidity.

## 2. Materials and Methods

### 2.1. Study Population and Sample Collection Burkina Faso

Blood samples were obtained from a cohort of 11 healthy young adults aged between 18 and 31 years in Nouna, an area in Burkina Faso characterized by high seasonal malaria transmission rates. The selected blood donors met specific criteria: (i) no fever episodes in the preceding 15 days, (ii) no vaccination in the past month, (iii) no medication intake within the last 2 weeks, and (iv) tested negative for HIV, hepatitis B virus (HBV), and syphilis (Venereal Disease Research Laboratory (VDRL) test). Blood donations were procured at the conclusion of the dry season, characterized by lower malaria transmission rates. Subsequently, sera were meticulously prepared through Ficoll (Merck) centrifugation and transported to Heidelberg for further analysis. Ethical approval for the study was obtained from the Ethical Committee of the Medical Faculty, University of Heidelberg (number 369/2003). Additionally, B. Kouyaté, the director of the Centre de Recherche en Santé de Nouna (CRSN), provided approval and support for the study.

### 2.2. Purification of Human Sera and PBMCs from Phase Ia Trial

A phase Ia clinical trial of full-length Plasmodium falciparum merozoite surface protein 1 (MSP1_FL_) was conducted between April 2017 and November 2018 in healthy naïve volunteers in Heidelberg (Germany) [36]. This first-in-human trial aimed at studying the safety and immunogenicity in a dose escalation manner of this vaccine candidate adjuvanted with the TLR4 agonist GLA-SE, an oil-in-water nanoemulsion glucopyranosyl lipid A. This study was randomized, double-blinded, placebo and adjuvant controlled. Blood was drawn from healthy volunteers (median age of 27.5 years (range 19–57), 81% of White Caucasian ethnic background). For serum preparation, S-Monovette serum gel vacutainers were used the serum was obtained from clotted blood by subjecting it to centrifugation for 10 min at 2500× *g*, followed by storage at −80 °C. For peripheral blood mononuclear cells (PBMC) isolation we employed vacutainer cell preparation tubes (CPT) with sodium citrate (BD Biosciences, San Jose, CA, USA). To isolate peripheral blood mononuclear cells (PBMCs), whole blood underwent centrifugation at 1700× *g* for 30 min. The PBMC layer obtained was carefully transferred and subjected to two washes with phosphate-buffered saline (PBS). The resulting pellet was then re-suspended in 0.5 mL of freezing medium, which consisted of either 50% fetal bovine serum (FBS) and 50% RPMI 1640 or 12.5% human serum albumin (HSA) and 87.5% RPMI 1640. The cell count was determined using trypan blue staining. Then, 0.5 mL of dimethyl sulfoxide (DMSO) medium was prepared (20% DMSO, 30% RPMI and 50% FBS) to rapidly freeze the cells. The DMSO medium was added gently and dropwise. The cells were first frozen at −80 °C and after 24 h, transferred for long-term preservation into liquid nitrogen. For the experiments, an anti-malaria human serum designated as the World Health Organization (WHO) reference reagent was obtained by pooling serum from individuals residing in Kimusu, Kenya. This pooling process was facilitated by the National Institute for Biological Standards and Control (NIBSC) under code 10/198. Importantly, these individuals had previous exposure to malaria, ensuring the relevance and efficacy of the reference reagent for the study [36]. Malawian adults’ purified pooled IgG was kindly provided by Prof. Dr. Faith Osier.

### 2.3. Enzyme-Linked Immunosorbent Assay (ELISA) for Anti-MSP1_FL_ IgG Subclasses

Enzyme-linked immunosorbent assay (ELISA) was employed to determine the titers of IgG subclass antibodies [36,38]. MaxiSorp plates (Thermo Fisher Scientific, Waltham, MA, USA) were coated with 0.1 mL coating buffer (34 mM Na_2_CO_3_ and 16 mM NaHCO_3_ at pH 10.6) containing 100 nM of the recombinant merozoite surface protein 1 (Glycotope Biotechnology GmbH, Heidelberg) [39,40]. Two-fold dilutions were used for sera titration. After 2 h of incubation, subclass-specific secondary antibodies were employed to detect binding antibodies. For the detection of IgG subtype antibodies, peroxidase-conjugated anti-human IgG1, anti-human IgG2, anti-human IgG3 and anti-human IgG4 antibodies (The Binding Site GmbH, Schwetzingen, Germany) were used, with dilutions set according to the manufacturer’s instructions. The substrate 1-step turbo tetramethylbenzidine (TMB) from Thermo Fisher was added for 20 min in the dark, followed by termination with 1 M HCl. Optical density (OD) readings were recorded at 450 nm. As a positive control, purified human myeloma protein (1 mg/mL, #BP078; BP080; BP082; BP084) diluted to 4 μg/mL in Tris-buffered saline (TBS) buffer at pH 8 was utilized as per the manufacturer’s instructions (The Binding Site GmbH).

### 2.4. Growth Inhibition Assay (GIA) for P. falciparum

*P. falciparum* 3D7 parasites were cultured in 4% hematocrit using a complete medium (Cmed) consisting of RPMI 1640 medium supplemented with 0.1 mM hypoxanthine, 20 μg mL^−1^ gentamycin and 0.25% Albumax I. Parasites were synchronized to schizont stages using 5% sorbitol; 25 µL/well of the parasite suspension adjusted to 0.6% parasitemia in Cmed was added to 25 µL/well of sera from vaccinees at days 0, 85, 210 and 365. Samples were dispensed in duplicates into 96-well U-bottom culture plates, each equipped with individual lids. Rabbit IgG targeting the *P. falciparum* apical membrane antigen 1 (AMA-1, 10 mg/mL) (purified IgG obtained from rabbits immunized with a mixture of 7 AMA-1 alleles and purified at BioGenes, Berlin, Germany) and WHO standard (Kenyan pool) served as the positive control. As the negative control, we employed a parasite control without antibodies. Uninfected erythrocytes were used as background signals. One complete parasite cycle (40–48 h) incubation period was then established in a gas chamber containing a tri-gas mixture (5% CO_2_, 5% O_2_, and 90% N_2_). The temperature was set at 37 °C. The next day, cold PBS was added to the wells, and the plates were frozen at −20 °C for >1 h as a lysing step. After thawing, each well was resuspended with pLDH (Plasmodium Lactate DeHydrogenase) substrate buffer containing 2 mg/10 mL of nitro blue tetrazolium (NBT), 50 μL of affinity-isolated IgG anti-dsDNA (APAD) and 200 μL of diaphorase for parasite quantification through the biochemical detection of pLDH. A wavelength of 650 nm was then used for determining the optical density (OD) using a Cytation 3 microplate reader. The inhibition percentage was calculated using the formula:

$$Inhibition\;=100-\frac{\left(A650\;serum\;sample-A650\;erythrocyte\;control\right)}{\left(A650\;parasite\;control-A650\;erythrocyte\;c\;control\right)}\times 100\%$$

### 2.5. Avidity Assay

To determine the avidity index for both total IgG and IgG subclasses, the same procedure as for the MSP1_FL_-specific ELISA assay previously published [36] was followed. For the total IgG avidity assay, an IgG titer normalization of all vaccinees’ serum samples tested for every timepoint [36] was previously conducted. Similarly, for the IgG subclass avidity assay, a previous IgG subclass titer normalization of all serum samples tested for every timepoint already described was also conducted. After the sera were added to the wells, 100 μL/well sodium thiocyanate (NaSCN) (Fulka #71938) in different concentrations ranging from 0–7 M was added for 15 min and incubated at room temperature (RT). After washing to remove antibodies bound with low avidity, we proceeded with the ELISA as described earlier for total IgG ELISA [36] and here described for IgG subclasses. A trendline with the ODs at 450 nm was created for every sample treated with 0–7 M of NaSCN. The avidity index for total IgG and IgG subclasses for all samples analyzed was calculated as the concentration [M] of NaSCN at which 50% of the ODs from the non-treated serum sample was obtained.

### 2.6. PBMCs Stimulation

Cryopreserved peripheral blood mononuclear cell (PBMC) samples were thawed in 1 mL of warm PBMC medium, which consisted of RPMI 1640, 10% fetal bovine serum, 200 mM L-glutamine and 1% penicillin/streptomycin. Additionally, 50 U/mL of Benzonase (Novagen Millipore GmbH, Darmstadt, Germany) was supplemented. After a wash, the cells were re-suspended in a warm PBMC medium, adjusted to a concentration of 10^6^ PBMCs/mL, and allowed to rest overnight at 37 °C in a 5% CO_2_, 5% O_2_ and 90% N_2_ environment. Cell numbers were determined using trypan blue staining and adjusted to 10^6^ cells/mL. Stimulation was initiated by adding 25 μg/mL MSP1_FL_. As a stimulation control, we used an anti-CD3 antibody (Mabtech #3605–1–50) at a final concentration of 0.1 μg/mL) and, furthermore, PBMC medium, for the unstimulated condition. For subsequent qPCR analysis, cells were collected and lysed after 16 h of stimulation. PBMCs intended for intracellular cytokine staining (ICS) analysis were harvested after 48 h of MSP1_FL_ stimulation. Supernatants designated for enzyme-linked immunosorbent assay (ELISA) analysis were collected after 24 h of MSP1_FL_ stimulation and stored at −20 °C until use.

### 2.7. Real-Time Reverse-Transcriptase (RT)-PCR

RNA extraction was carried out utilizing the RNeasy Mini-Kit (Qiagen, Crawley, UK) following the manufacturer’s guidelines. The protocol included optional on-column DNase digestion (RNase-free DNase set, Qiagen, Crawley, UK). For each condition and timepoint, 1 million cells were pelleted by centrifugation for 5 min at 300× *g*. We then removed the supernatant and cells were lysed with 350 μL RLT buffer. RNA was eluted in 30 μL RNase-free water. For the assessment of purity and quantity, we used the NanoDropTM Spectrophotometer (Thermo Fisher Scientific). Reverse transcription of 1 μg of RNA was performed using the Omniscript RT Kit (Qiagen), 0.5 μg Oligo(dT) primers (Thermo Fisher Scientific), 0.25 μM Random Nonamers (Sigma, Darmstadt, Germany) per assay and 40 U/μL RNase Inhibitor (RiboLock RNase Inhibitor, Thermo Fisher Scientific, Waltham, MA, USA) at a final concentration of 0.5 U/μL. RNA was denatured at 65 °C for 5 min and placed on ice until the final incubation with the Master Mix for 60 min at 37 °C. TaqManTM Fast Advanced Master Mix (Thermo Fisher Scientific, Waltham, MA, USA) was used for qPCR. An amount of 2 μL of the cDNA template was used for a final volume of 20 μL. Technical duplicates were employed for the IL-4 (Assay ID: Hs00174122_m1), the IL-10 (Assay ID: Hs00961622_m1) as well as the reference gene 18S rRNA (Assay ID: Hs99999901_s1) assays. The cDNA for 18S rRNA was diluted 20 fold before adding to the assay (0.1 μL of cDNA). For each plate and each RNA extract, NCT and no-RT controls were, respectively, included. Using a LightCyclerR 480 II (Roche Diagnostics, Indianapolis, IN, USA), the qPCR program included UNG activation at 50 °C for 2 min, polymerase activation at 95 °C for 20 s, 40 cycles of denaturation at 95 °C for 3 s and annealing at 60 °C for 30 s, with fluorescence measurement after every cycle. Cp calculation was performed with Abs Quant/2nd Derivative Analysis from LightCyclerR Software Version 1.5. Relative quantification was accomplished using the Pfaffl method [41], with an efficiency correction of 2 applied for each assay. Several reference genes were evaluated, leading to the selection of 18S rRNA as the most suitable reference gene for the application.

### 2.8. Flow Cytometric Analysis of Surface Markers and Intracellular Cytokines

To characterize MSP1_FL_-induced T-cells, 1 × 10^6^ unstimulated, anti-CD3 (Mabtech #3605-1-50)-stimulated (used as positive control) as well as MSP1_FL_-stimulated PBMCs (n = 3) were cultured for 48 h. Then, 16 h prior to flow cytometric analysis a protein transport inhibitor containing Brefeldin A (Cat # 555029, BD Biosciences) was added. PBMCs were stained in the dark for 30 min at 4 °C with antibodies specific to surface markers CD3 V500-C (Cat # 652896, BD Biosciences), CD4 PerCP-Cy™5.5 (Cat # 332772, BD Biosciences) and CD8 V450 (Cat # 560347, BD Biosciences). After fixing and permeabilizing, intracellular cytokine IFN-γ-FITC (Cat #340449, BD Biosciences) was applied to PBMCs for 30 min in the dark at 4 °C before flow cytometric analysis. To be sure, >1 × 10^5^ cells were acquired using BD FACSLyric and analyzed using FlowJo 10.2 or higher versions.

### 2.9. Enzyme-Linked Immunosorbent Assay (ELISA) for IL-4

The determination of IL-4 levels was conducted using the human IL-4 ELISA MAX^TM^ Standard Set Cat. No. 430301. Initially, the capture antibody was diluted in coating buffer (0.85 g NaHCO_3_, 3.56 g Na_2_CO_3_, pH 9.5 in 12 mL deionized water per plate). Subsequently, 100 μL of the capture antibody solution was added to all wells of a Nunc MaxiSorp 96-well plate, which was then sealed and incubated overnight at 4 °C. The following day, plates were subjected to four washes with 300 μL of wash buffer (TBS + 0.05% Tween-20) per well. All subsequent washes were performed similarly. To block non-specific binding and reduce background, 200 μL of assay diluent per well was added, and the plate was incubated at room temperature for 1 h with shaking on a plate shaker (500 rpm with a 0.3 cm circular orbit). All subsequent incubations with shaking were performed similarly. After washing the plate four times, 100 μL/well of standard dilutions and supernatants from PBMCs cultured for 24 h were added, and the plate was incubated for 2 h. Following another washing step, 100 μL of diluted detection antibody solution was added and incubated at room temperature for 1 h, followed by another washing step. Subsequently, 100 μL of diluted avidin–HRP solution was added to each well, and the plate was incubated at room temperature for 30 min, followed by another washing step. Then, 100 μL of TMB substrate solution was added and incubated in the dark for 30 min. The reaction was stopped by adding 100 μL of stop solution to each well, and absorbance was read at 450 nm within 15 min. Concentrations in pg/mL were estimated based on optical densities (ODs) by interpolating and extrapolating from the standard curve.

### 2.10. Statistical Analysis

Data analysis was conducted using Prism 9.3.1 (GraphPad) and R. The variations in titers of IgG subclasses, doses and avidity index between different timepoints were evaluated using the Friedmann test, followed by Dunn’s multiple comparison test. Additionally, Tukey’s multiple comparison test with Geisser–Greenhouse correction was applied where necessary. Pairwise correlations were computed using the non-parametric Spearman’s correlation. A significance level of *p* < 0.05 was considered statistically significant.

## 3. Results 

### 3.1. IgG Subclass Profile in Response to Repeated Immunization with MSP1_FL_

**Figure 2:**
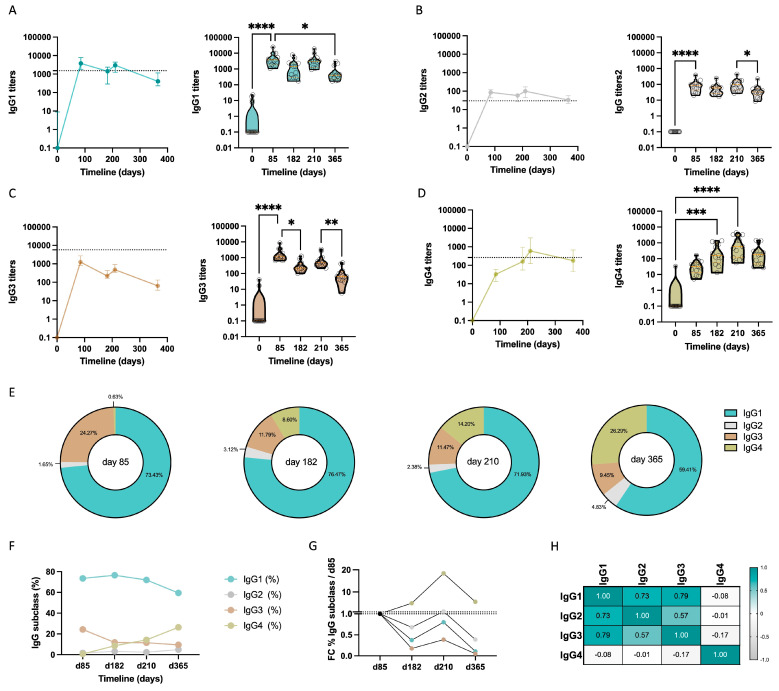
IgG3 to IgG4 shift after repeated immunization with MSP1_FL_. (**A**–**D**) Dynamics over the longitudinal study at days 0, 85, 182, 210 and 365 of titers of IgG subclasses IgG1 (**A**), IgG2 (**B**), IgG3 (**C**) and IgG4 (**D**). Titers represent the median ± IQR of a subcohort of 13 samples. Significant differences are shown in the violin plot. (**E**) Representative scheme of the titers proportion of every IgG subclass titer at each timepoint of the study. (**F**) Dynamics over the longitudinal study at days 0, 85, 182, 210 and 365 of the percentage of every IgG subclass titer. (**G**) Fold change of IgG subclass titers compared to day 85 over the longitudinal study at days 85, 182, 210 and 365. (**H**) Multiple correlation matrix showing correlation of all IgG subclasses calculated using Spearman order rank. *p* < 0.05 was considered statistically significant (* = *p* < 0.05; ** = *p* < 0.01; *** = *p* < 0.001; **** = *p* < 0.0001). Error bars represent median ± IQR.

We conducted a longitudinal study until the end of the study at day 365 to monitor the dynamics of anti-MSP1-specific IgG subclass responses after repeated immunization with MSP1_FL_. First, we characterized the IgG subclass profile of a representative subcohort of 13 volunteers who had received the complete 3 monthly immunizations, plus a booster (Figure 1A). Paired sera samples were analyzed at pre-immunization (day 0) and post-immunization at days 85, 182 (booster), 210 and 365. IgG1, IgG2, IgG3 and IgG4 titers were measured on a MSP1_FL_-specific and IgG subclass-specific ELISA and compared to the IgG subclass titers of pooled sera from semi-immune Kenyan adults. IgG1 titers from vaccinees increased significantly at day 85, one month after the third immunization (*p* < 0.0001), exceeding those from Kenyans throughout the whole study, except for day 365 in which a significant decrease was found compared to day 85 (*p* = 0.010) (Figure 2A and Supplementary Data S1). IgG3 titers also increased significantly after the third immunization (*p* < 0.0001) and after the booster (*p* < 0.0001), but never reached the level observed for the Kenyan reference levels (Figure 2C and Data S1) and significantly waned after the booster (*p* = 0.0081). IgG3 was the only subclass with a significant decrease after day 85 (*p* = 0.0126). Although at low levels, IgG2 titers from vaccinees also increased significantly after three immunizations (*p* < 0.0001) and even exceeded those of Kenyans at all analyzed timepoints (Figure 2B and Data S1). Interestingly, while IgG4 titers after three immunizations were still very low, the main increase was observed one month after the booster at day 210 (*p <* 0.0001) to exceed the IgG4 titers found for Kenyans and to later gradually wane, although not significantly (Figure 2D and Data S1). A representation of the proportionality of every IgG subclass at every timepoint and the dynamics and fold changes over the vaccination period is represented in Figure 2E–G. IgG1, IgG2 and IgG3 titers were highly correlated (*p <* 0.0001), with no correlation observed for IgG4 and the rest of the IgG subclasses (Figure 2H and Data S1).

To obtain a finer insight into the IgG subclass responses during the initial steps of the vaccination period, IgG1, IgG2, IgG3 and IgG4 optical density (OD) levels were determined between day 0 and day 85, including days 29 and 57, one month after the first and second immunization with MSP1_FL_ (Supplementary Figure S1 and Data S1). The degree of response for every IgG subclass varied at these initial timepoints. At day 29, one month after the first immunization, detectable levels of both IgG1 and IgG3 were already observed, while IgG2 levels were marginal and IgG4 undetectable. At day 57, one month after the second immunization, cytophilic IgG1 and IgG3 subclasses both increased significantly (*p* < 0.0001) to reach their highest level. At this timepoint, IgG2 and IgG4 were still very low. IgG2 levels slowly ramped up throughout the vaccination with a significant increase observed at day 85 (*p* < 0.0001). Of interest, IgG4 only progressed significantly (*p* = 0.0227) just over the limit of detection at day 85.

### 3.2. IgG Subclass Profile of Malaria Exposed Populations from Africa

**Figure 3:**
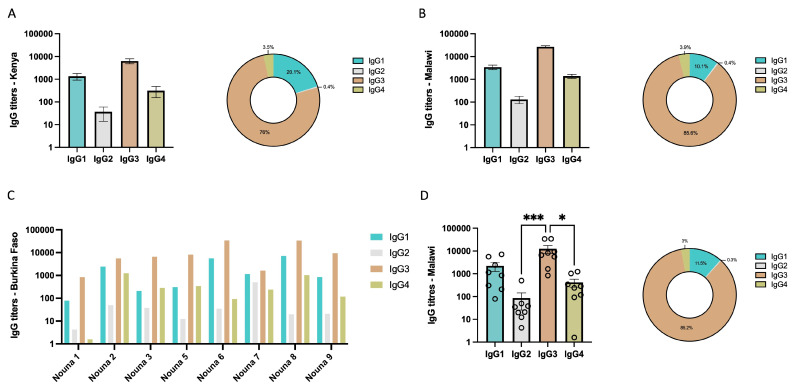
IgG titers from malaria-exposed populations from Africa. (**A**,**B**) IgG subclass titers and representative scheme of the titers proportion of (**A**) technical replicates (n = 5) of pooled sera from adult population from Kisumu, Kenya (WHO reference standard) and (**B**) technical replicates (n = 5) of purified IgG from sera from adult population from Malawi. (**C**) Individual IgG subclass titers of individuals from Nouna, Burkina Faso (n = 8). (**D**) Overall IgG subclass titers and representative scheme of the titers proportion of individuals from Nouna, Burkina Faso (n = 8). Error bars represent mean ± SD (**A**,**B**) or median ± IQR (**D**). Statistical differences between timepoints were calculated using Friedman test. *p* < 0.05 was considered statistically significant (* = *p* < 0.05; *** = *p* < 0.001).

Next, we compared the IgG subclass profile observed during MSP1_FL_ vaccination (Figure 2) with those from several malaria-exposed populations from Africa (Figure 3A–D and Data S2). Pooled sera from semi-immune Kenyan adults from Kisumu revealed high titers of IgG3, followed by IgG1 and moderate IgG4 titers. IgG2 titers were marginal (Figure 3A). A similar IgG subclass profile with high IgG3 titers was found when pooled IgG from Malawian adults was investigated (Figure 3B). To further test the heterogeneity of the IgG subclass profile, but this time within individuals, we analyzed serum samples (n = 8) from semi-immune individuals from Nouna, Burkina Faso. The repertoire of IgG subclasses varied between individuals (Figure 3C and Supplementary Figure S2). In general, high IgG3 titers governed the overall responses (mean = 6364.88, range = 4832.4–8621), followed at a distance by much lower titers of IgG1 (mean = 1349.18, range = 611.5–1692.6). IgG4 titers were in general low (mean = 315.299, range = 114–520.7), although some individuals showed exceeding levels (Nouna 2 and 8) (Figure 3D). IgG2 titers were in general the lowest (mean = 36.95, range = 21.84–77.7), with only higher titers observed for individual Nouna 7. No correlation could be found between the different IgG subclass titers and the individual’s age (Supplementary Figure S3). The overall IgG subclass picture of samples from Burkina Faso resembled that of Kenyans and Malawians with statistical differences observed between IgG3 and IgG2 (*p* = 0.0001 and *p* = 0.0117, respectively) (Figure 3D). In general, the main feature of African samples compared to MSP1_FL_ vaccinees was the high titers of IgG3, which we previously found to be lower and to vanish rapidly one month after the third immunization and the booster in vaccinees, whose preponderant IgG subclass was instead IgG1.

### 3.3. IgG4 Occurrence Is Weakly Correlated with Fc-Mediated Effector Functions

**Figure 4:**
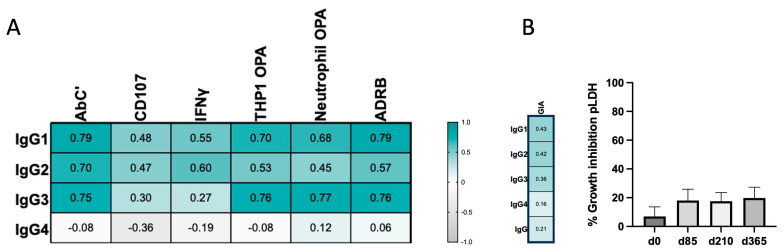
Correlation of IgG subclasses and Fc-mediated effector mechanisms. (**A**) Heatmap showing Spearman rank correlations between titers of IgG subclasses IgG1, IgG 2, IgG3 and IgG4 and Fc-mediated effector functions previously measured through in vitro assays [37]. The color intensity represents the strength of correlation. (**B**) Growth inhibition assay (GIA) of sera from vaccines (n = 4). The *P. falciparum* strain 3D7 was cultured for one cycle in the presence of total sera from volunteers pre-vaccination at d0 and after vaccination at d85, d182 and d365. The lack of correlation based on Spearman rank order between GIA and the titers of IgG subclasses IgG1, IgG2, IgG3 and IgG4 is also shown as a heatmap with the color intensity representing the strength of correlation. Error bars represent the mean ± SE.

It is widely accepted that Fc-dependent effector functions are mostly triggered by cytophilic IgG1 and IgG3 subclasses and that IgG2 and IgG4 subclasses have lower potential to mediate such antibody-driven functions. We have previously described the multifunctional nature of total IgG elicited after immunization with MSP1_FL_ and their dynamics [37] (shown schematically in Figure 1B). Our current findings of non-sustained IgG3, together with the significant increase in IgG4 at later timepoints in the immunization regimen, suggested exploring the correlation between IgG subclass titers dynamics and Fc-mediated effector mechanisms (Figure 4A and Data S3). Significant correlations were found for IgG1 titers with C1q complement fixation (Abc’) (*r* = 0.79, *p* < 0.0001), THP1-OPA (*r* = 0.70, *p* < 0.0001), neutrophil-OPA (*r* = 0.68, *p* < 0.0001), as well as for antibody-dependent respiratory burst (ADRB) (*r* = 0.79, *p* < 0.0001). Weaker correlations, although significant, were observed for NK-cell assays regarding degranulation (CD107a) (*r* = 0.48, *p* = 0.008) and IFNγ production (*r* = 0.55, *p* = 0.001). Similarly, for IgG3 titers, significant correlations were found with Abc’ (*r* = 0.79, *p* < 0.0001), THP1-OPA (*r* = 0.70, *p* < 0.0001), neutrophil-OPA (*r* = 0.68, *p* < 0.0001), as well as for ADRB (*r* = 0.79, *p* < 0.0001). For NK-cell assays, only CD107a was weakly correlated (*r* = 0.30, *p* = 0.045). IgG2 was also moderately correlated with all functions, although at a lower scale (*r* = 0.70 for Abc’, *p* < 0.0001; *r* = 0.53 for THP1-OPA, *p* = 0.0002; *r* = 0.45 for Neutro-OPA, *p* = 0.002; *r* = 0.57 for ADRB, *p* < 0.0001; *r* = 0.47 for CD107a, *p* = 0.001 and *r* = 0.60 for IFNγ, *p* < 0.0001). On the contrary, IgG4 titers did not significantly correlate with any functional assay. This suggests a role of the late increase in IgG4 titers in the loss of Fc-mediated effector functions described for total IgG at the end of the one-year follow-up period. 

In the context of malaria, neutralization represents the capacity of antibodies to block the invasion of parasites into red blood cells (RBCs). Contrary to Fc-mediated effector functions, neutralization is elicited in a Fab-dependent manner. To elucidate if the different IgG subclass profiles would lead to different neutralization capacities, we next assessed the neutralizing capacity using an in vitro growth inhibition assay (GIA). Only marginal (<20%) GIA activity was observed at all immunization timepoints, and thus no significant correlation could be assessed for any anti-MSP1-specific IgG subclass (Figure 4B).

### 3.4. IgG Avidity Increases after Vaccination and Is Associated with IgG4 Levels

**Figure 5:**
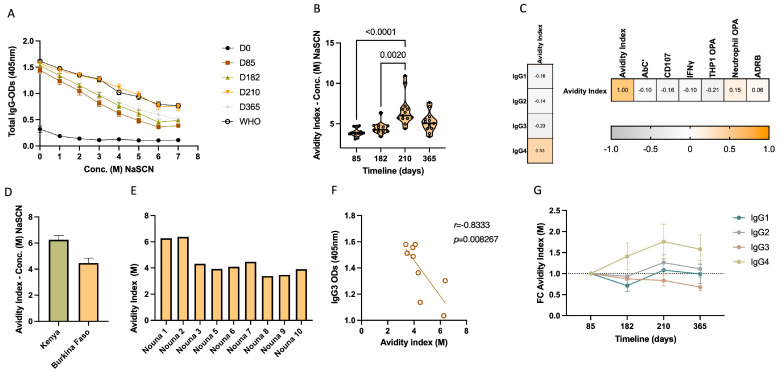
Increase in IgG4 is accompanied by an increase in avidity. (**A**) Different decreases in ODs in the IgG ELISA assay between different timepoints of the study and pooled sera from adult population from Kisumu, Kenya (WHO reference standard) tested, following a gradual increase in the concentration of NaSCN from 0 to 7 M. Error bar represents mean ± SE. (**B**) Total IgG avidity index calculated for d85, d182, d210 and d365 for samples (n = 13). Statistical differences between timepoints were calculated using Friedman test. (**C**) Heatmaps showing Spearman rank correlations between (i) the total IgG avidity index and the IgG1, IgG2, IgG3 and IgG4 titers and (ii) Fc-mediated effector functions previously measured through in vitro assays [37]. (**D**) Total IgG avidity index calculated for pooled sera from adult population from Kisumu, Kenya (WHO reference standard) and overall samples (n = 8) from Burkina Faso. Error bar represents mean ± SE. (**E**) Total IgG avidity index calculated for individual samples (n = 8) from adult population from Nouna, Burkina Faso. (**F**) Significant negative correlation calculated by Spearman rank order between the total IgG avidity index calculated for samples (n = 8) from Burkina Faso and the levels of IgG3. (**G**) Fold changes in the IgG subclass-specific avidity index at d85, d182, d210 and d365 (reference day 85) (n = 4). Slashed line represents the threshold of one-fold change. Error bars represent the mean ± SE.

We further investigated whether the delayed increase in the proportion of the IgG4 subclass would be accompanied by any other relevant functional consequence (Figure 5A–G and Data S4). To this end, paired sera from the same representative subcohort of 13 volunteers having received three immunizations, plus the booster were analyzed for their avidity index. The avidity index represents the concentration of a chaotropic agent, in this case, sodium thiocyanate (NaSCN), at which 50% of the IgG bound to an epitope/protein, in our case to the MSP1_FL_, is eluted off. We initially explored the total IgG avidity index throughout the longitudinal study by normalizing samples from different immunization timepoints to the same total IgG titer. In this assay, a gradual increase in the concentration of NaSCN from 0 to 7 M leads to decreasing ODs in the total IgG ELISA assay, meaning that less IgG remains bound to MSP1_FL_ coated on the plate (Figure 5A). Over the vaccination period, the total IgG avidity index increased gradually with significant differences being observed between the first timepoint at day 85 (median 3.83 M, range = 3.09–4.75 M) compared to day 182 (median 4.12 M, range = 3.39–6.39 M) and to the peaking avidity index observed at day 210 (median 5.65 M, range = 4.39–10.95 M) (*p* < 0.0001 and *p* = 0.0020, respectively). At day 365, the avidity index slightly dropped (median 5.05 M, range = 3.39–7.14 M) (Figure 5B). This total IgG avidity index dynamic resembled that of IgG4 titers and underscores the likely role of the IgG4 levels rising after day 85 in the late increase in avidity. Indeed, IgG4 was the sole IgG subclass whose titers moderately correlated with the avidity index (*r* = 0.53, *p* < 0.0001); yet, the avidity index did not correlate with Fc-mediated effector functions (Figure 5C). Pooled sera from semi-immune Kenyans showed an avidity index of 6.07 M, while sera samples from Burkina Faso (n = 9) had a mean of 4.47 M (Figure 5D), with a varying degree found inter-individually (range = 3.39–6.39 M) (Figure 5E). Of interest, the avidity index of samples from Burkina Faso correlated negatively with the highly multifunctional IgG3 (*r* = −0.7857, *p* = 0.0279) (Figure 5F). There was no correlation between the avidity index and the age of the individuals (Supplementary Figure S3).

To further explore the proposed association between IgG4 levels and avidity, an IgG subclass-specific avidity index assay was established. After normalizing the titers of all the IgG subclasses at different timepoints, the specific avidity index of IgG1, IgG2, IgG3 and IgG4 was calculated for a subset of vaccinees. We found that the avidity index (normalized to day 85) gradually increased for IgG4 from day 182 (median fold change = 1.56; range = 0.85–1.95) until day 210 (median fold change = 1.82; range = 1.57–2.55) and day 365 (median = 1.73; range = 1.36–2.22) (Figure 5G). Much lower fold changes were observed for IgG1 at day 210 (median fold change = 1.38; range = 0.86–2.02) and day 365 (median fold change = 1.17; range = 0.83–1.95), while no increases were observed for IgG2 and IgG3 (Figure 5G). 

### 3.5. Progressive Production of Interleukin-4 during Vaccination Parallels with the Late Increase in IgG4

**Figure 6:**
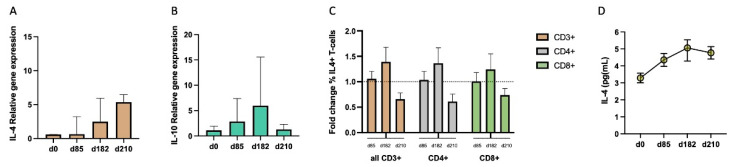
Interleukin-4 dynamic over the vaccination period. (**A**,**B**) Relative gene expression of non-inflammatory IL-4 (**A**) and IL-10 (**B**) as determined by qPCR after 16 h of stimulation of PBMCs with MSP1_FL_ was determined through the ΔΔCT method using 18s rRNA as reference gene and unstimulated control samples serving as background (n = 3). (**C**) Fold change in the percentage of IL-4^+^ producing CD3^+^ T-cells (brown), CD4^+^ T-cells (grey) and CD8^+^ T-cells (olive) after 48 h of stimulation of PBMCs with MSP1_FL_ determined by intracellular production of interferon gamma (IFN-γ) by flow cytometry using d0 as baseline. Slashed line marks the threshold of fold change one. (**D**) Fold change in the production of IL-4 during 24 h by cultured PBMCs and measured by ELISA (OD 450 nm) at days 0, 85, 182 and 210. Error bars represent median ± 95% CI (**A**,**B**) and mean ± SE (**C**,**D**).

Previous research on IgG subclasses suggests that the secretion of non-inflammatory cytokines by T follicular helper type 2 (Tfh-2) lymphocytes is implicated in the shift of balance from IgG subclasses towards IgG4. Hence, we investigated whether this could also be an underlying mechanism contributing to the described IgG4 shift in MSP_FL_ vaccinees (Figure 6A–D and Data S5). To test this hypothesis, we collected peripheral blood mononuclear cells (PBMCs) at day 0, 85, 182 and 210 from MSP1_FL_ vaccinees and stimulated them with MSP1_FL_ for 16 h before the expression levels of IL-4 and IL-10 were quantified by transcript-specific real-time Reverse Transcriptase (RT)-PCR. Although no major conclusions can be drawn due to the limited number available for testing, a gradual increase in the relative expression of IL-4 and IL-10 was observed after day 85 (Figure 6A,B). Consistent with higher transcriptional activity, intracellular staining (ICS) using flow cytometry also revealed a moderate increase in the percentage of IL-4^+^ T-cells of different subpopulations (total CD3^+^ T-cells, CD4^+^ T-cells and CD8^+^ T-cells) at day 182 (Figure 6C). We then analyzed the supernatants from cultured PBMCs stimulated with MSP1_FL_ and found a gradual cumulative increase in secreted IL-4 (Figure 6D).

## 4. Discussion

In the study presented here, we longitudinally tracked IgG subclass responses in volunteers vaccinated with MSP1_FL_ over a follow-up period of one year. Our findings suggest a reduction in cytophilic antibodies, especially IgG3, together with an expansion of the IgG4 subclass driven by repeated immunization with the MSP1_FL_ protein. Our results are in accordance with some previous reports regarding the proteinaceous VAX003 HIV vaccine [15], and what has been very recently experienced with mRNA vaccines against SARS-CoV-2 [15]. This phenomenon has become a subject of worry and debate and is raising important questions regarding its functional implications [41]. Encouraging for our MSP1_FL_-based vaccine is the finding that we observed a rapid increase in the cytophilic IgG1 and IgG3 antibody titers early on during vaccination (even shortly after the first immunization, at day 29) and that IgG1 represented the most abundant IgG subclass one year after the start of the trial. However, for most MSP1_FL_ volunteers in this trial, IgG3 titers never reached the high levels observed in several semi-immune adult populations from Africa during the study period. This is of great importance for the intrinsic capacity of these IgG subclasses to elicit Fc-mediated effector functions [26,27,28,29,30,31,32,33]. Fc-mediated effector functions were shown to correlate with protection in malaria-endemic populations [42,43,44]. Contrary to the leading blood-stage vaccine candidate RH5/AS01B, for which reduced blood-stage parasite growth rates in vaccinees following CHMI were associated with in vitro antibody-mediated GIA [45], antibodies generated by vaccination with MSP1_FL_ at the elicited titers in the naïve volunteers participating in this phase Ia do not lead to growth inhibition, with the potential implication in vaccine efficacy to be determined. Thus, the strength of our MSP1_FL_ vaccine candidate relies on the capacity to activate multiple Fc-mediated effector mechanisms, thus characterizing the dynamic of cytophilic IgG subclasses during the vaccination period is important. Future CHMI studies following vaccination with MSP1_FL_ will determine the impact of Fc-mediated effector functions on the challenge outcome.

Our study further revealed that small IgG4 levels were detectable only one month following the second immunization at day 57, but rapidly increased after the third immunization and the booster, to become the second most abundant anti-MSP1_FL_-specific IgG subclass at the end of the trial. High IgG4 responses have been rarely observed after infections [46], except for some parasitic infections such as *Brugia malayi*, with IgG4 representing even up to 90% of the IgG repertoire [47]. In allergies, IgG4 can also constitute more than 75% of allergen-specific IgG after continuous exposure to antigens [48]. IgG4, in particular, is regarded as an anti-inflammatory IgG, most probably due to Fab-arm exchange [49]. Moreover, IgG4 is associated with the development of tolerance [46]. For example, in beekeepers, venom-specific IgG4 slowly becomes the dominant IgG subclass [50] with time, whereas IgG1 appears during early exposure before their titer wanes. On chromosome 14, the order of the four γ heavy-chain genes within the immunoglobulin gene complex is γ3-γ1-γ2-γ4. Our results would support the hypothesis of consecutive events of sequential class switch recombination [51,52] through the activity of the activation-induced cytidine deaminase (AID) [53], from proximal IgG3 to distal IgG4, during the vaccination with MSP1_FL_.

IgG subclass switching post-vaccination has also been explored for other malaria vaccines. For example, the pre-erythrocytic malaria vaccine RTS,S leads to a pattern of IgG subclasses in which protection correlates with IgG1 and IgG3, while IgG2 and IgG4 are clearly underrepresented [35]. Nonetheless, some associations with protection were unexpectedly reported for IgG4 levels, but mainly to antigens not included in the vaccine construct such as MSP5 and MSP1, but not CSP [35]. As for the most advanced *P. falciparum* blood stage malaria vaccine RH5 [45], barely detectable IgG2 or IgG4 subclasses have been reported except for slight increases observed 270 days after the first immunization in the group immunized with the maximum antigen concentration (50 μg) [54]. Predominant IgG3 and IgG1 against the PfRH5 invasion complex have been reported to confer protection from *P. falciparum* malaria in natural infections [25].

Among the critical factors identified in other systems as potentially responsible for class switching to IgG4 are repeated vaccination and excessive antigen concentration [15,55]. Our data show a sharp increase in IgG4 levels already after the third immunization, which became detectable in all vaccinated individuals irrespective of whether they received a booster immunization. These findings align with an ongoing germinal center (GC) reaction throughout the vaccination period, suggesting that the appearance of IgG4 antibodies may result from consecutive events of class switch recombination and the expansion and maturation of IgG4-switched memory B cells. The levels of IgG4 from vaccinees after the third immunization, and, importantly, after the booster, are way over the titers observed for chronically exposed semi-immune adult populations from Kenya, Malawi and Burkina Faso. The phase Ia clinical trial of MSP1_FL_ involved dose escalation (25 μg, 50 μg or 150 μg of MSP1_FL_ protein), as well as a comparative study of three monthly immunizations with or without a booster. A weakness of our study is the limited number of samples available for the group without the booster immunization. This does not allow us to draw definitive conclusions on whether the number of immunizations is implicated as a catalyzer in the exaggerated IgG4 switch, although the profile of IgG subclasses over time seemed to follow the same pattern in the limited number of samples analyzed for the 3 immunizations group (Supplementary Figure S4 and Data S6). Experiments from other studies [15] involving repeated vaccination with either tetanus toxoid or respiratory syncytial virus infection do not support the notion of class switching to IgG4 being a general consequence of repeated antigen exposure in the form of vaccinations. Regarding the different doses of MSP1_FL_ administered, no significant differences were observed, except for immunization with 25 μg of MSP1_FL_, which led to higher levels of IgG4 at day 365 compared with 50 μg (Supplementary Figure S4 and Data S6), but this does not sustain for our vaccine the possibility of a more rapid switch towards IgG4 after higher doses administered [55]. Key for future clinical trials involving MSP1_FL_ will be to further investigate if the IgG subclass dynamics could favor IgG3 instead of IgG4 by testing lower vaccine doses administered in a new dose de-escalation study.

In our study, we have corroborated previous findings for SARS-CoV mRNA vaccines [15] of improved avidity and reduced Fc-mediated functionality of vaccine-induced IgG after repeated immunization, but in our case using a MSP1_FL_ protein vaccine. The gradual increase in avidity that we found was associated with a parallel increase in the levels of IgG4 by using a MSP1_FL_-specific and IgG subclass-specific ELISA assay. The administration of booster shots has also been proposed to significantly enhance the neutralizing capacity of antibodies [15]. This effect is attributed to the prolonged activation of GCs and the continuous maturation of B cells. The increase in avidity of IgG after the booster immunization with MSP1_FL_ did not result in GIA activity. The physiological ramifications of GIA activity are controversially discussed since GIA levels do not always correlate with protection in immunoepidemiological studies in malaria-endemic settings [44,56,57,58]. Prospective studies have pointed out that GIA activity is more present in young children and then wanes with increasing age [59,60,61,62,63,64], in parallel with higher expression of IgG4 levels in consequence of a lifetime repeated exposure to infections through mosquito bites [65]. Future studies will determine if the IgG subclass dynamics that we report here vary when the vaccine is tested in younger populations.

The vaccine kind may play a role in class switching to IgG4. For example, the IgG4 subclass does not prevail after repeated vaccination with tetanus toxoid or respiratory syncytial virus infection [15]. Furthermore, IgG4 antibodies are not induced after vaccination with adenoviral vectors against SARS-CoV-2 [15]. These findings may encourage the exploration of alternative MSP1_FL_-based vaccination platforms in particular adenovirus vectors. Moreover, in the case of a proteinaceous vaccine, alternative adjuvants preventing IgG4 induction would be desirable [66]. The GLA-SE adjuvant employed in our vaccine is a stable oil-in-water nanoemulsion of the TLR4 agonist glucopyranosyl lipid A. GLA-SE was chosen for its favorable safety record [67,68,69] and because it stimulates Th1 responses to co-administered antigens [70] known to contribute to protective immunity to blood-stage malaria infection [71]. Nonetheless, our data suggest that our vaccine also stimulates Th2 responses, which have been reported to promote functional antibodies *during P. falciparum* infection [72], as the ones elicited by vaccination with SumayaVac-1 [36,37], and potentially implicated in the increase in IgG4. Testing the effect of other available adjuvants mediating protection against malaria through distinct immune mechanisms in future studies will be very valuable for global vaccination strategies against malaria.

Studies on various viral pathogens, such as influenza viruses, RSV, and SARS-CoV-2, have shown that different antibody functions mediated through Fc receptors contribute to varying degrees of protection [73,74,75,76]. Similar results have been reported in the malaria field [28,29,30,33,77,78]. IgG4 has been described as possessing limited potential to exert strong functionality for Fc-dependent effector functions such as phagocytosis as well as complement activation [27,79]. In our previous first-in-human study of SumayaVac-1, we observed a reduction in the functionality of total IgG after the third immunization with MSP1_FL_, especially after the booster [37]. This was tested through a battery of antibody-mediated in vitro assays involving different immune cell players such as neutrophils, monocytes and NK cells, as well as complement fixation [37]. When we correlated these effector functions at every timepoint with the different IgG subclasses, IgG1, IgG3, and intriguingly IgG2, were correlated with these Fc-mediated mechanisms, whilst IgG4 was the only IgG subclass not correlated. IgG2 and IgG4 are believed to outcompete the cytophilic IgG1 and IgG3 in terms of antigen recognition and binding, thereby preventing or inhibiting potentially harmful long-term inflammatory processes [80,81]. 

## Data Availability

The authors declare that the data supporting the findings of this study are available within the article and its Supplementary Information Files or are available from the authors upon request. Requests for materials should be addressed to R.T.-L.

## Supplementary Materials

The following supporting information can be downloaded at: https://www.mdpi.com/article/10.3390/vaccines12020208/s1, Figure S1: Early dynamics of IgG subclasses; Figure S2: IgG3 is the preponderant IgG subclass of individuals from Nouna, Burkina Faso; Figure S3: Lack of correlation between the age of individuals from Nouna, Burkina Faso and their titers of IgG subclasses and avidity; Figure S4: Similar IgG subclass dynamics regarding number of immunizations and dose administered. Data S1; Data S2; Data S3; Data S4; Data S5; Data S6.
