# Supplementary material for: IgG Subclass Switch in Volunteers Repeatedly Immunized with the Full-Length Plasmodium falciparum Merozoite Surface Protein 1 (MSP1)
Source: Vaccines (Basel). 2024 Feb 17;12(2):208. doi: 10.3390/vaccines12020208 (PMC10893298; doi:10.3390/vaccines12020208)
Supplement: Supplementary file 1 [file vaccines-12-00208-s001.zip › vaccines-2862941-supplementary.pdf]

vaccines

Article

IgG Subclass Switch in Volunteers Repeatedly Immunized with the Full-Length *Plasmodium falciparum* Merozoite Surface Protein 1 (MSP1)

Veronika Rathay [1], Kristin Fürle [1], Viktoria Kiehl [1], Anne Ulmer [1], Michael Lanzer [1] and Richard Thomson-Luque [1,2,*]

[1] Parasitology, Centre for Infectious Diseases, University Hospital Heidelberg, Medical Faculty, Heidelberg University, 69120 Heidelberg, Germany
[2] Sumaya-Biotech GmbH & Co. KG, 69115 Heidelberg, Germany

Supplementary Material

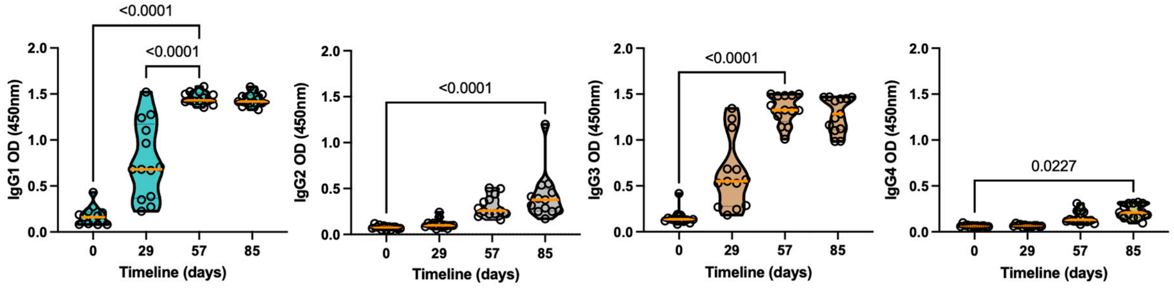

Supplementary Figure S1. Early dynamics of IgG subclasses. Levels of IgG1, IgG2, IgG3 and IgG4 were measured at days 0, 29, 57 and 85. Violin plots represent median ± IQR of a subcohort of 13 samples. $p < 0.05$ was considered as statistically significant. (* = $p < 0.05$; ** = $p < 0.01$; *** = $p < 0.001$; **** = $p < 0.0001$).

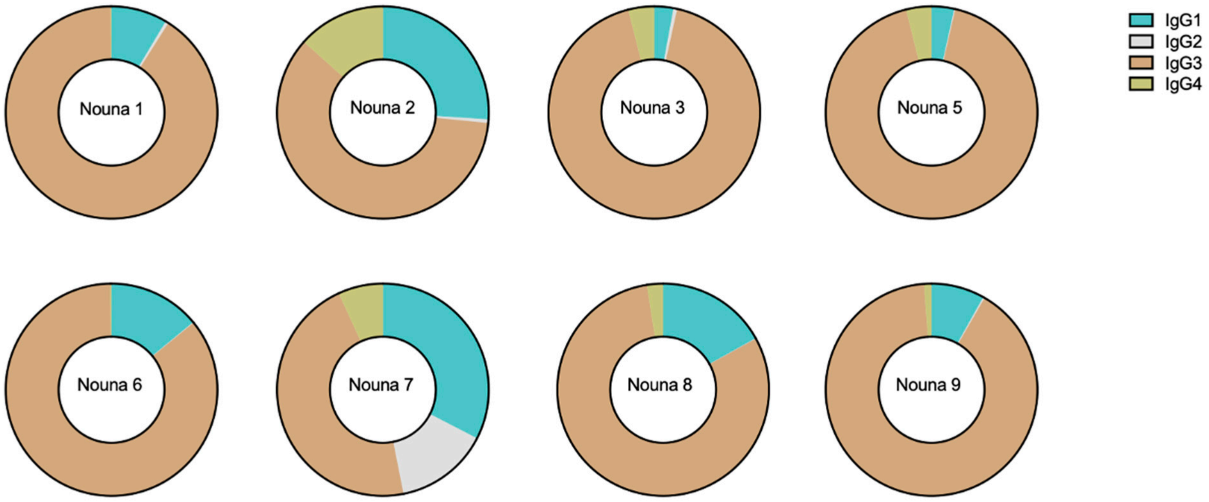

Supplementary Figure S2. IgG3 is the preponderant IgG subclass of individuals from Nouna, Burkina Faso. Representative scheme of the titers proportion of every IgG subclass titer showing interindividual differences in IgG1 (green), IgG2 (grey), IgG3 (brown) and IgG4 (olive).

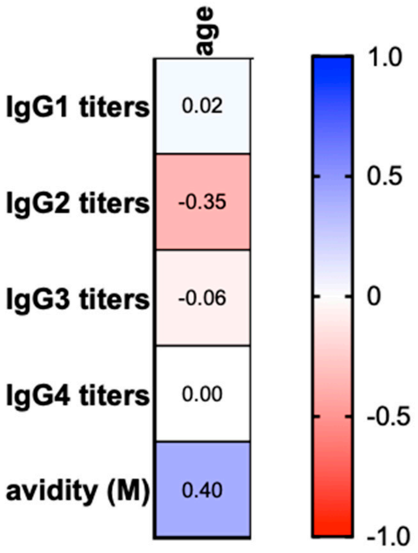

Supplementary Figure S3. Lack of correlation between the age of individuals from Nouna, Burkina Faso and their titers of IgG subclasses and avidity. Heatmap showing spearman rank correlations between age of the individuals with the IgG1, IgG2, IgG3 and IgG4 titers as well as with the total IgG avidity index measured.

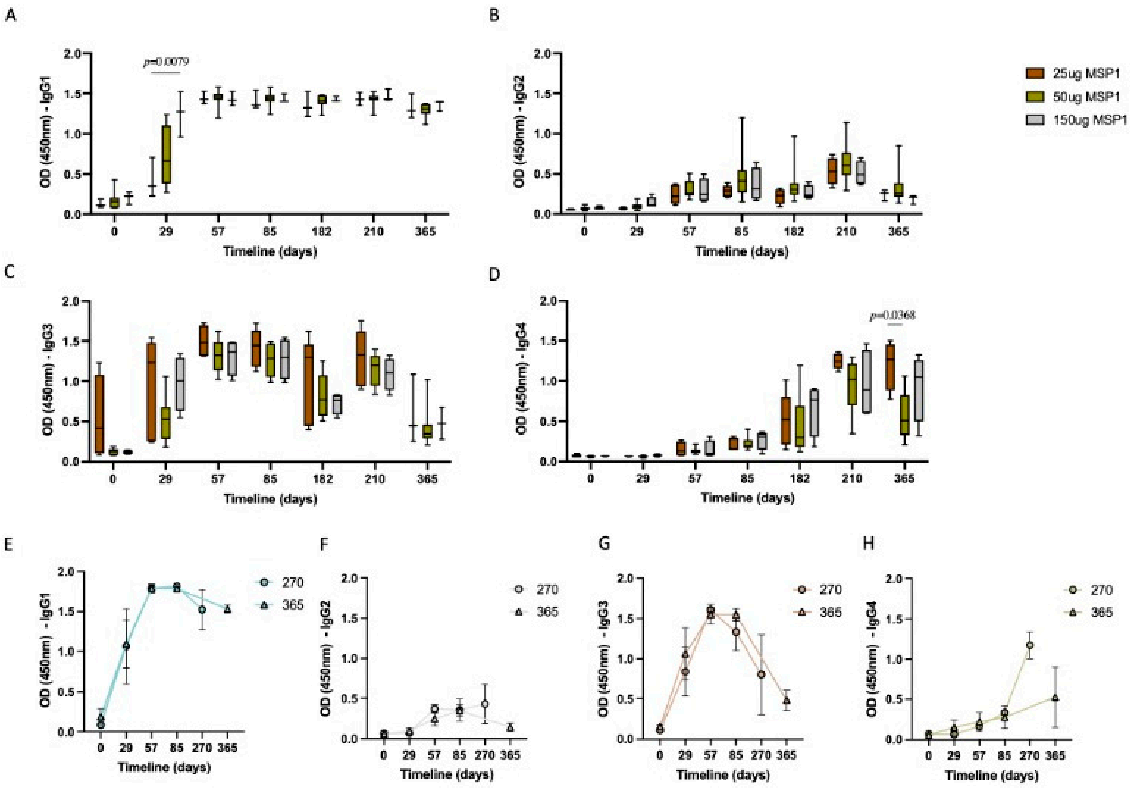

Supplementary Figure S4. Similar IgG subclass dynamics regarding number of immunizations and dose administered. (A-D) IgG subclass levels of vaccinees immunized with 25 µg (brown) 50 µg (olive) and 150 µg (grey), measured by ELISA for IgG1 **(A)**, IgG2 **(B)**, IgG3 **(C)** and IgG4 **(D).** Statistical differences between timepoints were calculated using Tukeys multiple comparison test with Geisser-Greenhouse correction. **(E-H)** IgG subclass levels of vaccinees immunized with 3 monthly doses of $MSP1_{FL}$ and followed up until day 270 post first vaccination (n = 2 volunteers receiving 50 µg of the vaccine) or day 365 post first vaccination (n = 3 volunteers receiving either 25 µg (n = 1) or 150 µg (n = 2)): **(E)** IgG1, **(F)** IgG2, **(G)** IgG3 and **(H)** IgG4. Box and whiskers show minimum and maximum values. Error bars represent the mean ± SE.
